# Melanoma Inhibitory Activity and regional lymph node status in malignant melanoma patients

**DOI:** 10.1186/1479-5876-13-S1-P11

**Published:** 2015-01-15

**Authors:** Angela Sandru, Silviu Voinea, Eugenia Panaitescu, Madalina Bolovan, Adina Stanciu, Sabin Cinca, Alexandru Blidaru

**Affiliations:** 1 Department of Surgical Oncology, “Carol Davila” University of Medicine and Pharmacy; “Alexandru Trestioreanu” Oncologic Institute, Bucharest, Romania; 2 Department of Medical Informatics and Biostatistics ,“Carol Davila” University of Medicine and Pharmacy, Bucharest, Romania; 3 Department of Carcinogenesis and Molecular Biology, “Alexandru Trestioreanu” Oncologic Institute, Bucharest, Romania

## Background

For many cancers, and malignant melanoma (MM) makes no exception, regional lymph node (RLN) status is decisive for subsequent patients’ outcome. But particularly for MM, stage III comprises a very heterogeneous group of patients, from those with microscopic invasion diagnosed by sentinel node (SN) biopsy to those with matted nodes. Correct staging of patients with clinically free RLN requires SN biopsy. Because this is an invasive maneuver, various substitutes were searched. Thus, in literature there are conflicting references to a possible link between Melanoma Inhibitory Activity (MIA) serum concentration and SN status [1,2]. MIA, a protein secreted by malignant melanocytes into the extracellular space, blocks the melanoma cells attachment to fibronectin and laminin. Thus MIA increases malignant cells mobility and promotes local invasion and metastasis [3]. In this context, we sought a connection between RLN status and MIA serum concentration in our group of patients.

## Materials and methods

150 patients with non-metastatic cutaneous MM were treated in our clinic between 2009 and 2013. They were staged according to 2009 AJCC (American Joint Cancer Committee) staging system. 47 patients were assigned to stage III: 37 presented with clinically evident lymphadenopathies and 10 had positive SN. SN biopsy was performed in 61 patients with intermediate thickness MM and no clinical or ultrasound signs of RLN metastasis. MIA was measured preoperatively in all patients. A cut-off value of 9.4 ng/mL was calculated using ROC curve [4]. Patients were divided in four groups according to RLN status (N0/N1/N2/N3).

## Results

Mean and median MIA serum concentration progressively increased along with the number of metastatic RLN. The difference between mean and median MIA values in the 4 groups was not accidental, but the consequence of different tumor load (p = 0.001). Only N0 patients had mean and median MIA concentrations less than 9.4 ng/mL, for all the other N categories, values surpassing the threshold. SN identification rate was 100%. The mean and median MIA serum value in SN positive group were higher than in SN negative one, but the differences were not statistically significant (p=0.6191) and in both cases didn`t exceed the upper normal limit.

## Conclusion

MIA serum concentration increases with RLN tumor burden. In our study SN status didn`t correlate with MIA value, but the small number of patients prevents us to draw a conclusion.
